# A Comprehensive Exploration of the Biological Effects of Adipose-Derived Stem Cells in the Treatment of Systemic Sclerosis

**DOI:** 10.3390/cells14060458

**Published:** 2025-03-19

**Authors:** Gabriele Storti, Riccardo Foti, Roberta Foti, Marco Palmesano, Martina Patacchiola, Dalila Incognito, Giulio Cervelli, Benedetto Longo, Maria Giovanna Scioli, Elena Fiorelli, Sonia Terriaca, Andrea Lisa, Bong Sung Kim, Augusto Orlandi, Valerio Cervelli

**Affiliations:** 1 Plastic Surgery, Department of Surgical Sciences, University of Rome “Tor Vergata”, 00133 Rome, Italy; gabriele.storti@uniroma2.it (G.S.); marcopalmesano@gmail.com (M.P.); martinapatacchiola@gmail.com (M.P.); benedetto.longo@uniroma2.it (B.L.); valeriocervelli@virgilio.it (V.C.); 2 Division of Rheumatology, A.O.U. “Policlinico-San Marco”, 95123 Catania, Italy; robertafoti@hotmail.com; 3 Department of Medicine and Surgery, University of Enna “Kore”, 94100 Enna, Italy; 4 PhD Program in Applied Medical Surgical Sciences, Department of Surgical Sciences, University of Rome “Tor Vergata”, Viale Oxford 81, 00133 Rome, Italy; andrealisamd@gmail.com; 5 Medical Oncology Unit, Department of Human Pathology “G. Barresi”, University of Messina, 98122 Messina, Italy; dalilaincognito96@gmail.com; 6 Department of Experimental Medicine, University of Rome “Tor Vergata”, 00133 Rome, Italy; giulio.cervelli@gmail.com; 7 Anatomy Pathology Institute, Department of Biomedicine and Prevention, University of Rome “Tor Vergata”, 00133 Rome, Italy; scioli@med.uniroma2.it (M.G.S.); elena.fiorelli1@gmail.com (E.F.); terriacasonia093@gmail.com (S.T.); orlandi@uniroma2.it (A.O.); 8 Department of Plastic and Reconstructive Surgery, European Institute of Oncology, IRCCS, 20139 Milan, Italy; 9 Department of Biomedical Sciences, Humanitas University, 20072 Milan, Italy; 10 Department of Plastic Surgery and Hand Surgery, University Hospital Zurich, 8006 Zurich, Switzerland; bong-sung.kim@usz.ch

**Keywords:** systemic sclerosis, ADSC, lipofilling

## Abstract

Systemic sclerosis (SSc) is a complex autoimmune disease characterized by vasculopathy and tissue fibrosis affecting the skin and internal organs. Genetic and environmental factors influence susceptibility, severity, and onset. Current treatments are limited and not always effective, leading researchers to investigate new approaches, such as the use of adipose-derived mesenchymal stem cells (ADSCs) through fat grafting. This review seeks to understand how ADSCs may impact the development and progression of SSc, with a particular focus on how these cells could alter immune responses and reduce fibrosis. ADSCs have been found to affect various immune cells, including T cells, B cells, macrophages, and dendritic cells, by releasing cytokines, chemokines, and growth factors. These interactions generally suppress inflammation and promote a regulatory immune environment. Additionally, ADSCs can influence the extracellular matrix, helping to prevent fibrosis through signaling molecules like exosomes. ADSCs show promise as a treatment for SSc due to their ability to modulate the immune system and reduce fibrosis. Early clinical studies are encouraging, but more research is needed to fully understand how they work and to develop effective treatment protocols.

## 1. Introduction

Systemic sclerosis (SSc) is a chronic autoimmune disorder with complex pathogenesis involving fibrosis and vascular abnormalities.

The annual incidence of SSc is estimated to be below 10 per 100,000 individuals in both regions, ranging from 0.6 to 2.3 per 100,000 individuals in Europe and 1.4 to 5.6 per 100,000 individuals in North America. Notably, there has been a trend of increasing reported incidence rates over time, potentially due to enhanced disease recognition, improved diagnostic techniques, and heightened awareness among healthcare providers [1].

Significant variability in the reported prevalence of systemic sclerosis (SSc) has been observed, with slightly higher estimates in North America (13.5–44.3 per 100,000 individuals) compared to Europe (7.2–33.9 per 100,000 individuals). This difference may reflect genuine regional epidemiological variation or could be attributable to differences in clinical data collection and analysis methodologies.

Adipose-derived stem cells (ADSCs) are emerging as a promising therapeutic option for SSc due to their regenerative, immunomodulatory, and anti-fibrotic properties. Unlike bone marrow-derived mesenchymal stem cells (BM-MSCs), ADSCs are accessible with minimal invasiveness, making them a favorable candidate for regenerative therapies [2]. ADSCs are a subset of mesenchymal stem cells with the ability to differentiate into multiple cell types, including chondrocytes, osteocytes, myocytes, and adipocytes. Additionally, ADSCs demonstrate the capacity to differentiate in vitro into cells from other germinal lineages, such as endothelial cells and neurons. Their wide-ranging clinical applications include the treatment of chronic wounds, breast and esthetic surgeries, burns, osteocartilaginous reconstruction, and autoimmune diseases such as systemic sclerosis [3]. Furthermore, they have a high paracrine capacity, which helps in stimulating angiogenesis, immunomodulation, and reducing fibrosis. These effects together may be beneficial in the treatment of the manifestations of SSc. In clinical settings, ADSCs have primarily been utilized through fat grafting, a procedure in which the lipoaspirate is processed and reinjected into the fibrotic areas. Another option is the isolation and injection of the stromal vascular fraction (SVF), the perivascular component containing ADSCs, endothelial cells, and other regenerative cell types [4,5,6]. This approach has shown promising results in improving skin elasticity, reducing fibrosis, and enhancing overall skin quality in SSc patients, particularly in commonly affected areas such as the face and hands. Studies report that fat grafting provides structural support through adipocytes, while ADSCs and endothelial cells support blood vessel regeneration and collagen remodeling, potentially reversing some effects of SSc-associated tissue fibrosis [7,8,9,10,11,12].

Due to the abundant availability of fat tissue, the ease of harvest via liposuction, and the high concentration of stem and stromal cells compared to bone marrow, fat grafting and adipose-derived cell therapies are emerging as promising treatments for SSc, particularly for addressing fibrosis and ischemic complications [13].

This review was conducted through a comprehensive literature search in databases such as PubMed and Scopus, focusing on studies evaluating the therapeutic potential of ADSCs in systemic sclerosis. References were selected based on their relevance, study design, and clinical significance, prioritizing peer-reviewed articles and recent findings. Our aim was to provide an overview of the current evidence while highlighting key advances and areas requiring further investigation. Indeed, the aim of this review was to provide a comprehensive exploration of the biological effects of ADSCs in systemic sclerosis, analyzing their immunomodulatory, anti-fibrotic, and pro-angiogenic properties, as well as their potential clinical applications. By summarizing current evidence and highlighting the gaps in knowledge, we seek to offer insights into the therapeutic potential of ADSCs and directions for future research.

## 2. Systemic Sclerosis (SSc)

SSc is a complex autoimmune disease characterized by a combination of vasculopathy, immune dysregulation, and tissue fibrosis affecting both the skin and internal organs. Its development is influenced by genetic predisposition and environmental factors [14]. The condition occurs predominantly in females, with a 6:1 ratio compared to males, although males tend to experience more severe disease manifestations and higher mortality rates. The peak age of onset is between 30 and 60 years [15].

SSc is classified into two main subsets based on the extent of skin involvement and associated systemic features. Diffuse cutaneous systemic sclerosis (dcSSc) involves skin thickening that extends proximal to the elbows and knees or affects the thorax and abdomen. This form is associated with a higher risk of internal organ damage, including interstitial lung disease, renal crisis, and cardiac complications, and is generally linked to a worse prognosis due to its aggressive nature. Limited cutaneous systemic sclerosis (lcSSc) involves skin thickening confined to areas distal to the elbows and knees, such as the hands, feet, and forearms. This subset is more commonly associated with pulmonary arterial hypertension and gastrointestinal complications and typically has a milder course with a better overall prognosis [16].

### 2.1. Pathogenetic Mechanisms

Systemic sclerosis (SSc) is marked by progressive fibrosis due to the accumulation of extracellular matrix components in various tissues and organs. Other hallmarks include specific autoantibodies, such as centromere (ACA), topoisomerase 1, and RNA polymerase III (RNApol3), along with vascular damage and inflammation [17].

The involvement of both adaptive and innate immune systems is a key aspect of SSc pathogenesis [18]. Vascular injury is thought to trigger the heightened immune response observed in the disease. This interaction between vascular damage and immune activity is complex and potentially reciprocal. Immune cells release fibrogenic factors, including the transforming growth factor (TGF)-β, interleukin-13 (IL-13), and interleukin-4 (IL-4), which are elevated in SSc patients. Experimental models suggest that inhibiting these factors can reduce fibrosis. IL-4 and IL-13, produced primarily by type 2 helper T (Th2) cells, promote B cell growth, immunoglobulin secretion, and the expression of adhesion molecules [19]. Additionally, these cytokines increase the tissue inhibitors of metalloproteinases (TIMP)-1, suppress enzymes like MMP1 and MMP3, and enhance TGFβ production, fibroblast activity, and extracellular matrix (ECM) synthesis [20]. TGFβ plays a central role in the progression of SSc by activating pathways involved in both fibrosis and inflammation. It phosphorylates SMAD proteins to drive SMAD4 expression and activates the mitogen-activated protein kinase (MAPK) pathway, involving JNK, p38, and ERK1/2. TGFβ also induces IL-13 production through the transcription factor GATA-3, creating a feedback loop that sustains immune cell and fibroblast activation [21]. Both innate and adaptive immunity contribute to SSc, though pinpointing the primary cells driving disease initiation remains a challenge. The disease is associated with M2 macrophage infiltration and monocyte activation in the affected skin. Unlike inflammatory M1 macrophages, M2 macrophages support tissue repair and excessive ECM deposition. Both macrophage subtypes release pro-inflammatory mediators like IL-6 as well as fibrogenic cytokines such as TGFβ, IL-4, and IL-13 [22]. Neutrophils also play a significant role in SSc by generating reactive oxygen species (ROS), which stimulate fibroblasts and promote fibrogenic cytokines like TGFβ, IL-6, and VEGF. Neutrophils from SSc patients produce more neutrophil extracellular traps (NETs) than normal, contributing to immune activation through interferon-α production and plasmacytoid dendritic cell (pDC) stimulation [23].

### 2.2. Clinical Manifestations

SSc can present different clinical manifestations involving several body districts that can potentially be the target of ADSC-based treatments. Ocular involvement is a significant yet often under-recognized feature of SSc. Common manifestations include dry eye disease due to tear gland fibrosis, reduced choroidal thickness reflecting systemic microvascular changes, eyelid abnormalities from skin tightening, retinal vascular changes, increased intraocular pressure, and cataracts [24]. Indeed, dry eye syndrome is prevalent in SSc patients, with significant involvement of the meibomian glands leading to lipid tear dysfunction. It is characterized by reduced tear production, increased osmolarity, and inflammation, correlating with disease severity and duration. Early ophthalmologic evaluation is essential for managing ocular manifestations and improving patient outcomes [25]. Moreover, SSc shows retinal vascular damage detectable with OCT-A, including reduced vessel density and retinal thinning, reflecting systemic microvascular impairment [26]. These ocular complications can impair vision and quality of life, particularly if left untreated. SSc treatment strategies involve immunomodulation, vascular modulation, and symptom management, tailored to individual disease manifestations. Immunomodulatory therapies, such as mycophenolate mofetil (MMF) and cyclophosphamide, target fibrotic and inflammatory pathways, particularly in interstitial lung disease (ILD). MMF has emerged as a first-line agent due to its superior safety profile compared to cyclophosphamide. Tocilizumab, targeting IL-6, and rituximab, targeting B cells, have shown efficacy in stabilizing lung function and reducing skin fibrosis [27]. Nintedanib, an anti-fibrotic agent, is effective in slowing lung function decline in ILD but does not address skin fibrosis. Vascular complications, including Raynaud’s phenomenon and pulmonary arterial hypertension (PAH), are managed with vasodilators like phosphodiesterase inhibitors, endothelin receptor antagonists, and prostaglandin analogs. These are often combined to treat PAH and enhance outcomes. Despite advances, significant unmet needs remain, particularly in early disease intervention and personalized treatment strategies [28].

Digital ulcers (DUs) in SSc are among the most debilitating complications of the disease, resulting from severe vasculopathy and impaired microcirculation. The pathogenesis of DUs is complex, involving a combination of endothelial injury, structural vascular remodeling, and persistent ischemia. In SSc, endothelial dysfunction is driven by immune-mediated damage and an imbalance between vasoconstrictive factors (e.g., endothelin-1) and vasodilatory mediators (e.g., nitric oxide), leading to progressive microangiopathy and reduced peripheral blood flow [29]. This ischemia predisposes patients to ulcer development, particularly in high-risk areas such as the fingertips and extensor surfaces. DUs are often a sign of advanced vascular disease, with microangiopathy playing a pivotal role. Capillary loss and structural remodeling, evident through nailfold capillaroscopy, are strong predictors of ulceration. These vascular changes are frequently exacerbated by local mechanical factors, such as trauma or calcinosis, which further impair skin integrity. Macrovascular involvement may also contribute, as evidenced by ulnar artery occlusions and reduced perfusion in patients with severe disease [30]. The heterogeneity of DUs is highlighted by their classification. Ischemic ulcers arise primarily from compromised microcirculation, while calcinosis-associated ulcers result from calcium deposits breaching the skin, often leading to secondary infections. Gangrenous ulcers represent the most severe form, characterized by critical ischemia and necrosis, which may necessitate amputation. The Amanzi classification system categorizes ulcers as ischemic, calcinosis-related, or gangrenous, providing a structured approach to treatment and prognosis. Studies indicate significant variation in healing times based on etiology, with ischemic ulcers generally resolving more rapidly than calcinosis- or gangrene-associated ulcers [31]. On a molecular level, DU pathogenesis involves platelet activation and impaired fibrinolysis, promoting microthrombus formation and further compromising tissue perfusion. This ischemic cascade is worsened by persistent vasoconstriction and reduced neoangiogenesis, which impede healing. Inflammation plays a dual role, aiding initial wound repair but aggravating tissue damage in chronic ulcerative states. Notably, infections, particularly with pathogens like Staphylococcus aureus, complicate the clinical course, delaying healing and increasing the risk of osteomyelitis or sepsis [32]. Advances in imaging modalities, such as laser Doppler imaging and nailfold capillaroscopy, have improved our understanding of the microvascular changes underlying DUs. These tools enable the early identification of at-risk patients and facilitate the monitoring of therapeutic responses. Capillaroscopic findings, including the “late” pattern with avascular areas and giant capillaries, are strongly associated with DU severity and outcomes [33]. The pathogenesis of DUs underscores the need for an integrated vascular diagnostic approach in SSc. Therapeutic strategies targeting both microvascular and macrovascular dysfunction hold potential for modifying disease progression. Current treatments aim to enhance perfusion using vasoactive agents like prostanoids and phosphodiesterase inhibitors, alongside addressing autoimmunity and optimizing wound care to prevent complications such as infection and gangrene [34].

**Figure 1:**
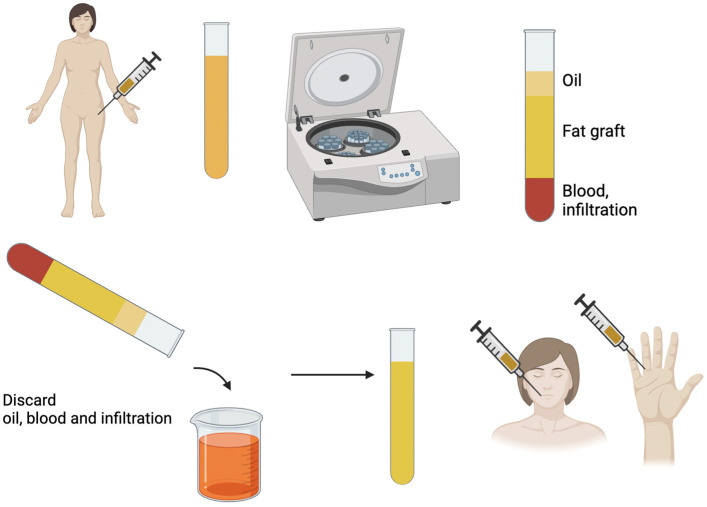
The Coleman technique involves the harvesting of adipose tissue, centrifugation, and the removal of the oily portion, as well as the part containing blood and local anesthetic. The fat graft is then injected into the recipient areas.

Another critical and debilitating feature of the disease is perioral fibrosis, characterized by excessive collagen deposition in the perioral tissues. This pathological process leads to microstomia (reduced oral aperture), perioral furrowing, and lip retraction, which significantly impair essential functions such as eating, speaking, and maintaining oral hygiene. Approximately 70% of patients with SSc exhibit head and neck involvement, including microstomia, which is often defined as an interincisal distance of less than 30 mm. These manifestations not only hinder functionality but also profoundly affect facial esthetics, contributing to social and psychological burdens for affected individuals [35]. Pathogenetically, perioral fibrosis arises from the interplay of immune dysregulation, microvascular dysfunction, and persistent fibroblast activation. The fibroblasts in SSc exhibit constitutive activation driven by molecular mediators such as transforming growth factor-beta (TGF-β), connective tissue growth factor (CTGF), and other cytokines, resulting in unchecked collagen production and reduced degradation. This fibrotic process is exacerbated by the characteristic microvascular changes in SSc, including capillary rarefaction and reduced tissue perfusion, which further fuel the cycle of ischemia and fibrosis [36,37]. The clinical consequences of perioral fibrosis extend beyond functional limitations. The tightness and thickening of the skin around the mouth produce the characteristic “purse-string” appearance, accompanied by radial perioral folds and nasal ala atrophy. These changes profoundly alter facial expressions and esthetics, significantly impacting quality of life. Affected patients often report difficulties in mastication, phonation, and basic oral hygiene, leading to secondary complications such as periodontal disease and malnutrition [35,38]. Therapeutic options for perioral fibrosis are evolving, with autologous fat grafting emerging as a promising intervention. This technique leverages the regenerative potential of ADSCs, which exhibit anti-fibrotic properties through angiogenic, immunomodulatory, and anti-apoptotic effects. These cells also influence fibroblast activity, promoting extracellular matrix remodeling and enhancing tissue elasticity. Studies utilizing the Coleman method for fat grafting have shown improvements in perioral pliability, oral aperture, and esthetic outcomes, reflected in reduced scores on the Mouth Handicap in Systemic Sclerosis (MHISS) Scale [39] (Figure 1).

Early intervention with fat grafting appears particularly beneficial, as it may prevent severe deformities by counteracting the progression of fibrosis. This is consistent with findings that ADSCs reduce fibrosis through mechanisms involving the modulation of the TGF-β pathway and vascular enhancement [40,41]. Complementary approaches, such as fractional CO_2_ laser treatments, have also demonstrated efficacy in addressing microstomia by increasing oral aperture and reducing radial perioral folds. These therapies provide additional tools for mitigating the functional and esthetic impacts of perioral fibrosis, suggesting a potential integrative strategy for comprehensive care [35,42]. Despite these advancements, further research is needed to optimize therapeutic protocols. Key variables, such as the ideal graft volume, the timing of interventions, and long-term outcomes, require clarification through larger, multicenter trials. Additionally, the synergistic effects of combining regenerative therapies like fat grafting with physical rehabilitation or laser treatments warrant exploration to develop a standardized, effective treatment framework for this complex condition [36,43].

Beyond the oral and vascular complications discussed earlier, SSc frequently affects the hands, which are particularly vulnerable to the disease’s fibrotic and vascular processes. This involvement is not only common but also among the most debilitating aspects of SSc, significantly impacting daily activities and overall quality of life. Indeed, hand involvement in SSc affects up to 97% of patients and is characterized by progressive skin thickening, tendon fibrosis, and joint contractures, which significantly impair mobility and dexterity [44]. Management strategies focus on mitigating these disabling features and improving hand function. Conventional approaches, including vasodilators and physical therapy, provide limited relief. Innovative regenerative therapies, such as fat grafting, are emerging as promising treatments. In randomized trials, ADSC injections have shown significant improvements in hand mobility, pain reduction, and the healing of digital ulcers, with sustained benefits over time [45]. Despite these advancements, challenges remain in standardizing protocols and evaluating long-term outcomes. Further research is needed to refine these approaches and expand their accessibility to patients suffering from severe hand involvement in SSc [46].

## 3. ADSCs

ADSCs are a type of mesenchymal stem cell (MSC) found in adipose tissue, in the perivascular niche. Derived from the stromal vascular fraction (SVF) of adipose tissue, ADSCs can be easily harvested through minimally invasive procedures such as liposuction, making them an attractive alternative to other MSC sources like bone marrow [47,48].

ADSCs possess several defining features, including their ability to self-renew and differentiate into multiple cell types, such as adipocytes, osteocytes, chondrocytes, and myocytes. They exhibit surface markers similar to other MSCs, including CD73, CD90, and CD105, while lacking hematopoietic markers such as CD34, CD45, and CD14. Furthermore, ADSCs secrete a variety of bioactive molecules, including cytokines, growth factors, and extracellular vesicles, which contribute to their immunomodulatory and anti-inflammatory effects [49].

The high abundance of ADSCs in adipose tissue—estimated to be 100–500 times greater than in bone marrow—makes them a particularly accessible and efficient source of stem cells for therapeutic applications [50].

There are many possible applications of ADSCs that are currently being investigated, such as regenerative medicine, immunomodulation, esthetic medicine, neurological disorders, and cancer therapy [51].

ADSCs can be isolated from adipose tissue through a systematic process involving harvesting, enzymatic digestion, and cell separation. Tissue is typically collected via minimally invasive lipoaspiration under local or general anesthesia, with common donor sites including the abdomen and thighs [52]. The harvested lipoaspirate is processed to remove contaminants and then digested enzymatically with collagenase to release cells from the extracellular matrix and obtain the SVF. The cell pellet containing the SVF, which includes the ADSCs, is resuspended in culture medium and seeded on Petri dishes. ADSCs are then cultured under sterile conditions for expansion and defined by a position statement of the International Society for Cellular Therapy (ISCT). Three minimal criteria define cultured MSCs: (i) plastic adherence in standard culture conditions; (ii) positivity for the expression of CD105, CD73, and CD90 and negativity for CD45, CD34, CD14 or CD11b, CD79α or CD19, and HLA-DR surface molecules; (iii) potential to undergo trilineage differentiation (adipogenic, chondrogenic, and osteogenic) [53,54].

### 3.1. Possible Biological Mechanisms Underlying ADSC Effects in SSc

**Figure 2:**
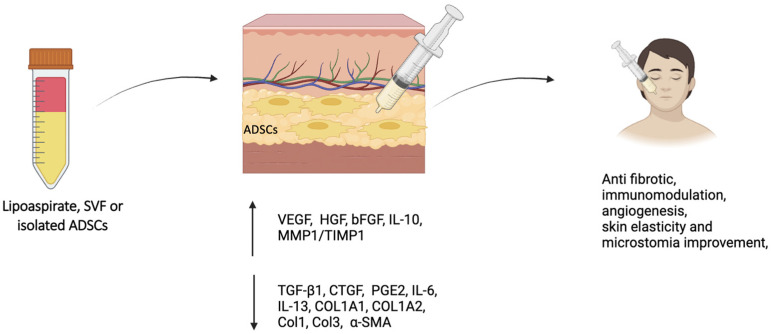
The injection of the adipose-derived stem cells (ADSCs) in the skin areas affected by systemic sclerosis (SSc) acts through different biological pathways, which ultimately determine multiple clinical effects. Abbreviation: SVF (stromal vascular fraction).

ADSCs show potential in reducing fibrosis in systemic sclerosis (SSc) by using various mechanisms: they secrete bioactive molecules that shift the immune response from pro-inflammatory to anti-inflammatory, reducing cytokine levels and promoting regulatory T cell formation [55]; they inhibit myofibroblast activation and reduce collagen production by downregulating the TGF-β/Smad pathway [56]; they enhance angiogenesis by releasing factors like VEGF and HGF, improving blood flow and reducing ischemic damage [57]; and they release extracellular vesicles containing microRNAs that target profibrotic pathways [58] (Figure 2).

#### 3.1.1. Regulation of Inflammatory and Immune Responses

ADSCs play a dual role in SSc by actively suppressing inflammation through the secretion of factors like the vascular endothelial growth factor (VEGF), basic fibroblast growth factor (bFGF), and interleukin-10 (IL-10), which collectively contribute to reducing fibrosis and encouraging angiogenesis. Additionally, ADSCs have been observed to inhibit T-cell proliferation and alter T-helper cell differentiation, reducing the inflammatory response and promoting a regulatory immune environment. These effects highlight ADSCs’ potential to address not only fibrosis but also the immune dysregulation characteristic of SSc [59].

ADSCs exhibit strong immunomodulatory effects on T lymphocytes, even offering potential in immunotherapy [60]. Primarily, ADSCs suppress T cell proliferation, especially under conditions of excessive immune activation, as seen in autoimmune diseases and organ transplants. They release anti-inflammatory cytokines, such as IL-10 and TGF-β, which help reduce inflammation by directly impacting T cell behavior. Additionally, ADSCs inhibit T cell differentiation into pro-inflammatory effector cells like Th1 and Th17, mitigating chronic inflammation [61].

Kuca-Warnawin E et al. examined the capacity of ADSCs from patients with SSc to modulate T-cell activation markers. The researchers aimed to determine if ADSCs from SSc patients retain immunomodulatory functions, particularly the ability to influence T-cell activation, despite the inflammatory and autoimmune background of the disease. ADSCs were isolated from SSc patients and compared to those from healthy donors. Both untreated and cytokine-preactivated ADSCs were co-cultured with allogeneic peripheral blood mononuclear cells (PBMCs) and activated CD4+ T cells. Findings revealed that SSc-derived ADSCs maintained their immunomodulatory effect, downregulating CD25 and HLA-DR expression while upregulating CD69 on both CD4+ and CD8+ T cells, similar to healthy donor-derived ADSCs. This modulation occurred primarily through soluble factors rather than direct cell-to-cell contact. Notably, however, SSc-derived ADSCs had a reduced inhibitory effect on CD25 expression when co-cultured with autologous T cells compared to allogeneic cells, suggesting that T cells from SSc patients may exhibit some resistance to ADSC-mediated immunosuppression [62].

ADSCs can also effectively inhibit T-cell proliferation. Researchers used ASCs from healthy donors and SSc patients to evaluate their effects on T-cell subsets, co-culturing them with PBMCs or purified CD4+ T cells under both direct cell-to-cell contact and transwell conditions. The anti-proliferative effects of ASCs were predominantly mediated through soluble factors, with kynurenines and prostaglandin E2 (PGE2) being the key mediators, while direct contact played a minor role.

Results indicated that all ADSCs, including those from SSc patients, significantly decreased T-cell proliferation, demonstrating their preserved immunomodulatory function despite the inflammatory background of the disease. However, ADSCs from patients exhibited slightly reduced inhibitory effects on CD4+ T cells compared to those from healthy donors. Inhibiting kynurenine and PGE2 pathways diminished the anti-proliferative effect, confirming their critical role. TGFβ did not influence the ADSCs’ function, while IL-10 contributed slightly to the inhibition only in SLE-ADSCs [63].

Similarly, another study demonstrated that ADSCs from patients with SSc impact T-helper cell differentiation, focusing on Th1, Th2, Th17, and regulatory T (Treg) cell subsets. Researchers used ASCs from both healthy donors and patients, co-culturing them with CD4+ T cells and PBMCs to assess their immunomodulatory potential. They measured transcription factors and cytokine levels associated with each Th subset to elucidate ADSCs’ effects.

The findings showed that ADSCs from both patient groups shifted the T-helper cell response from a Th1 to a Th2 profile, marked by a reduction in the IFNγ/IL-4 ratio, and facilitated the formation of Th17 and Treg cells. This modulation was primarily through influencing the expression of lineage-specific transcription factors, with a notable upregulation of Th2-related GATA3 and Th17-associated RORc in the presence of ADSCs. SLE and SSc ADSCs exhibited some transcriptional deviations, especially in Th1 and Treg lineage genes, but these differences did not alter their capacity to direct the immune response, suggesting that ADSCs from autoimmune contexts retain substantial regulatory functions in Th-cell differentiation [63].

Immunohistochemical staining revealed that ADSCs decrease the infiltration of CD4+ and CD8+ T cells, as well as macrophages, into the skin of the bleomycin-model mice. Additionally, the real-time PCR analysis of skin samples demonstrated a reduced mRNA expression of fibrogenic cytokines, such as IL-6 and IL-13, along with lower levels of type I collagen (COL1A2) in ASC-treated mice. The flow cytometry of spleen cells indicated that ASCs also reduced the frequency of IL-6-producing effector B cells and cytokine-producing CD4+ T cells (including IL-13 and IL-17 producers) in the bleomycin model [64].

#### 3.1.2. Reduction in Fibrosis

ADSCs exert anti-fibrotic effects through paracrine signaling, as demonstrated by co-culturing ADSCs from SSc patients with SSc fibroblasts. These co-cultures showed reduced fibroblast viability, proliferation, and the decreased expression of fibrotic mediators such as TGF-β1 and connective tissue growth factor (CTGF) [65].

These cells secrete vascular endothelial growth factor (VEGF), basic fibroblast growth factor (bFGF), and interleukin-10 (IL-10), which collectively modulate fibroblast activity, reducing excessive collagen deposition and promoting a more regulated extracellular matrix turnover. Recent studies, including Tanaka et al. (2022), have highlighted the role of mesenchymal stem cells in fibroblast regulation, demonstrating their ability to balance tissue remodeling processes in fibrotic conditions [66].

A study conducted by Maria A.T. et al. provided evidence that ADSCs possess considerable anti-fibrotic potential for SSc, demonstrating their ability to mitigate fibrosis in both skin and lung tissues in a preclinical model. ADSCs are significantly more effective than BM-MSCs in reducing fibrosis, as evidenced by decreases in skin thickness, collagen deposition, and the expression of fibrotic markers (Col1, Col3, and α-SMA) in both skin and lung tissues. The potent anti-fibrotic effect of ADSCs is associated with a reduction in inflammatory cytokines (TNF-α and IL-1β) and an increase in the MMP1/TIMP1 ratio, which favors extracellular matrix remodeling. Importantly, neither ADSCs nor BM-MSCs are found to migrate long term to injured tissues, suggesting that their anti-fibrotic effects are primarily mediated through paracrine signaling rather than direct tissue integration. This supports the potential of ADSCs as a more potent anti-fibrotic cell therapy option compared to BM-MSCs for treating SSc [67]. ADSCs promote the secretion of anti-fibrotic factors and matrix metalloproteinases within the grafted fat. Additionally, the fat grafts appear to enhance capillary networks in the fibrotic areas, further supporting tissue remodeling [40]. Current research on the therapeutic effects of lipotransfer in reversing fibrosis in systemic sclerosis focuses on the role of ADSCs in modulating the fibrotic pathway. Three key mechanisms under investigation include the regulation of transforming growth factor beta-1 (TGFβ1), the promotion of angiogenesis, and the modulation of the immune response [68]. TGF-β1 is a key driver of collagen synthesis, and its dysregulation can lead to fibrosis. In a bleomycin-induced fibrotic murine model, Chen et al. found that ADSC treatment reduced skin thickness and hydroxyproline content compared to controls. The ADSC group also exhibited lower TGF-β1 levels and increased vascular endothelial growth factor (VEGF) levels [69].

Strategies have been proposed to optimize ADSC-based therapies for SSc by modulating cell properties through pharmacological interventions. For example, Suzuka T. et al. investigated the enhanced therapeutic potential of adipose-derived mesenchymal stem cells (ADSCs) activated with low-molecular-weight heparin (LMWH) in the treatment of fibrosis. Using a bleomycin (BLM)-induced SSc mouse model, researchers assessed whether LMWH treatment could improve ASC functionality, specifically their migration ability and hepatocyte growth factor (HGF) secretion. The study found that LMWH-activated ASCs (hep-mASCs) exhibited significantly enhanced migration to affected skin tissues and higher HGF secretion than untreated ASCs. Histological evaluations revealed that hep-mASC-treated mice showed notable reductions in skin thickness and collagen content, accompanied by a decreased expression of key fibrotic markers, such as α-smooth muscle actin (α-SMA), TGF-β1, and collagen type I alpha 1 (COL1A1). Additionally, inflammatory cytokines, including interleukin-2 (IL-2) and interleukin-17 (IL-17), were also downregulated, indicating both anti-fibrotic and anti-inflammatory effects. The effects were more substantial with higher LMWH concentrations, implying that LMWH not only amplifies ASCs’ regenerative potential but also strengthens their migratory and anti-inflammatory properties [70].

#### 3.1.3. Pro-Angiogenic Effect of ADSCs

**Figure 3:**
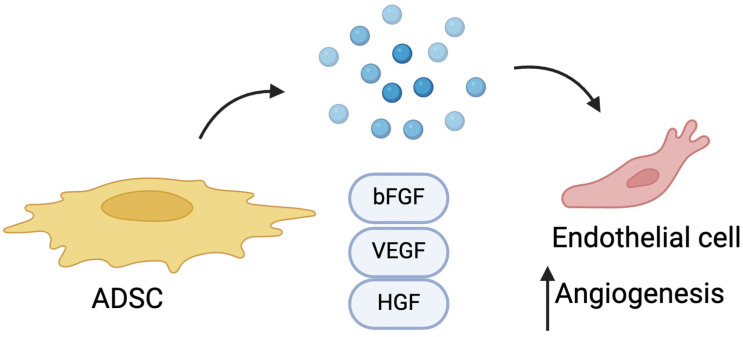
ADSCs exert their pro-angiogenic role by releasing VEGF, HGF, and bFGF, which stimulate angiogenesis.

The pro-angiogenic effect of ADSCs primarily stems from their secretion of growth factors and pro-angiogenic cytokines, such as vascular endothelial growth factor (VEGF), hepatocyte growth factor (HGF), and basic fibroblast growth factor (bFGF). (Figure 3). These secreted factors support neovascularization by stimulating nearby cells and tissues, enhancing blood vessel formation. The efficacy of ADSCs in ischemia models is mainly attributed to this paracrine signaling rather than direct cell replacement. Furthermore, ASCs can differentiate into endothelial cells, contributing directly to angiogenesis and vascular repair [71].

VEGF promotes the formation of new blood vessels, thereby improving vascularization in affected tissues. This effect is particularly beneficial in scleroderma, where microvascular damage leads to tissue ischemia and damage. The pro-angiogenic properties of ADSCs help enhance oxygenation and nutrient delivery to tissues, reducing fibrosis and improving skin elasticity and functionality. Furthermore, through their paracrine action, ADSCs modulate inflammation and support tissue regeneration [72,73].

To investigate the pro-angiogenic potential, Velier M. et al. conducted a study where ADSCs were co-cultured in transwell inserts with human microvascular endothelial cells (HMVEC-dA). This setup enabled the evaluation of angiogenic activity through tube formation assays on Matrigel and 3D spheroid sprouting assays. In the spheroid assay, while HMVECs co-cultured with SSc-ADSCs exhibited slightly shorter sprouts than those with healthy donor ADSCs, both ADSC types maintained a substantial capacity to promote angiogenic sprouting, indicating that SSc-ADSCs still support endothelial cell function. For the anti-fibrotic evaluation, ADSCs were co-cultured with dermal fibroblasts (DFs) isolated from SSc patients, using indirect transwell models to examine the paracrine effects on fibrosis markers. After five days of co-culture, Western blot analyses revealed significant reductions in α-smooth muscle actin (αSMA) and collagen content within the fibroblasts, indicating anti-fibrotic effects driven by the ADSC secretome. Additionally, qRT-PCR and multiplex assays were employed to quantify relevant factors in the ASC secretome, including VEGF-A, MMP-2, TIMP-1, HGF, and TGFβ1, showing no major differences between ADSCs from SSc patients and healthy donors [74].

#### 3.1.4. ADSC-Derived Extracellular Vesicles

Extracellular vesicles (EVs) derived from ADSCs could be a potential cell-free option for SSc treatment. In one study by Rozier et al., EVs were shown to be more effective than the cells themselves in exerting anti-fibrotic effects, significantly reducing fibrosis markers such as α-SMA and COL1A1 while enhancing remodeling markers like MMP1/TIMP1 [75].

Rozier P. et al. demonstrated that the ability of ADSCs to alleviate skin and lung fibrosis in a murine model of SSc is linked to miR-29a-3p, a microRNA present in the EVs, which targets genes associated with fibrosis, remodeling, and apoptosis. Both small (ssEVs) and large EVs (lsEVs) effectively halted disease progression in the HOCl-induced SSc model. Notably, the downregulation of miR-29a-3p in EVs abolished the therapeutic effects. miR-29a-3p regulates key genes, including Dnmt3a, Pdgfrbb, and Bcl2, influencing methylation, apoptosis, and fibrosis. Furthermore, EVs derived from human ASCs exhibited comparable efficacy to murine MSC-EVs [58,76].

The capabilities of EVs and their microRNAs have also been demonstrated in cardiac fibrosis. Specifically, EVs transport key microRNAs, such as miR-29 and miR-24, which play a crucial role in promoting cardiac repair by effectively inhibiting fibrosis and apoptosis [77].

### 3.2. Possible Effects of SSc on ADSCs

The phenotypical and functional characteristics of ADSCs derived from SSc patients have been studied extensively to evaluate their potential for therapeutic use. Research indicates that ADSCs from SSc patients retain a phenotype similar to that of healthy donor-derived ADSCs, with comparable surface marker profiles (e.g., CD73, CD90, and CD105) and the ability to differentiate into adipogenic, osteogenic, and chondrogenic lineages. Despite maintaining fundamental stem cell characteristics, disease-specific alterations in proliferation, metabolism, and the functional properties of ADSCs from SSc patients suggest challenges for their therapeutic efficacy in autologous applications [78].

Although ADSCs are frequently used in the treatment of conditions secondary to SSc [79], research has shown that ADSCs can be significantly influenced by the patient’s pathological conditions. In particular, various inflammatory states have been shown to promote the onset of a senescent phenotype in ADSCs. Similarly, diseases such as systemic sclerosis have been observed to alter the normal differentiation capabilities of ADSCs, potentially compromising their regenerative potential. In 2019, an Italian group of researchers demonstrated that serum from systemic sclerosis (SSc) patients can drive the differentiation of normal adipose-derived stem cells (ADSCs) toward a profibrotic myofibroblast phenotype, instead of allowing them to undergo normal adipogenesis. In this in vitro model, ADSCs treated with SSc serum showed reduced adipogenic markers (like perilipin and adiponectin) and upregulated myofibroblast markers (including α-SMA, S100A4, and type I collagen). Additionally, these ADSCs acquired a contractile function, confirmed through collagen gel contraction assays, similar to those induced by TGF-β1. Elevated TGF-β1 levels in SSc serum were hypothesized to play a major role in this phenotypic shift, suggesting that the pathological environment in SSc may promote profibrotic ADSC behavior, thereby contributing to tissue fibrosis in SSc [80].

Taki Z. et al. examined how the SSc microenvironment influences ADSCs, potentially activating them into pathogenic myofibroblast-like cells. Using patient-derived blister fluid (BF) from early-stage diffuse SSc, researchers treated cultured ADSCs from healthy donors to model the SSc environment’s impact on these cells. Exposure to SSc BF led ADSCs to adopt a profibrotic phenotype, with an increased expression of α-smooth muscle actin (αSMA), type I collagen, and connective tissue growth factor (CTGF), markers characteristic of myofibroblasts. Pathway analysis implicated TGF-β as a significant driver of this activation, with additional involvement from lactate and mechanical cues within the SSc microenvironment. Mechanosensing pathways, including RhoA and integrin-linked kinase (ILK), were upregulated, highlighting the influence of extracellular matrix stiffness [81].

ADSCs from SSc patients exhibit a reduced proliferation rate, diminished metabolic activity, and decreased capacity to adhere to extracellular matrix components. These functional impairments may limit their ability to home to damaged tissues or effectively integrate into fibrotic areas. Furthermore, the exposure of ADSCs to the SSc microenvironment, characterized by high levels of TGF-β and other profibrotic factors, induces the expression of fibrosis-associated markers, including α-smooth muscle actin (α-SMA), type I collagen, and connective tissue growth factor (CTGF). This response suggests that ADSCs in SSc patients might be primed toward a profibrotic phenotype, potentially exacerbating fibrosis under certain conditions by differentiating into myofibroblast-like cells [82].

Molecular studies further support the idea that the pathological environment in SSc influences MSCs. An analysis of microRNA (miRNA) profiles in MSCs derived from ADSCs and bone marrow (BM-MSCs) in SSc patients reveals distinct miRNA expression patterns associated with profibrotic pathways, particularly those involving the TGF-β signaling pathway. While both AD-MSCs and BM-MSCs share a profibrotic miRNA signature, specific miRNAs implicated in cellular senescence, proliferation, and extracellular matrix remodeling differ based on the tissue origin. These findings suggest that the SSc microenvironment primes MSCs from various sources toward a fibrotic phenotype. Targeting miRNA dysregulation has emerged as a potential avenue for therapeutic intervention to modulate fibrosis-associated pathways in SSc-MSCs [83].

Despite these challenges, ADSCs retain significant immunomodulatory properties even in the context of SSc. The systemic infusion of autologous culture-expanded ADSCs has been shown to reduce inflammatory cytokine levels and support immune regulation, promoting a balanced immune response. In SSc patients, ADSCs have demonstrated the capacity to alleviate tissue damage associated with fibrosis by modulating the immune environment, thereby supporting tissue integrity and reducing autoimmune-driven fibrotic processes. Additionally, when co-cultured with endothelial cells, ADSCs from both SSc patients and healthy donors exhibit pro-angiogenic potential, supporting tube formation and enhancing angiogenesis under hypoxic conditions [84].

Overall, these findings underscore the complex interplay between the disease environment and ADSC function in SSc. While ADSCs from SSc patients retain many key properties, including immunomodulation and pro-angiogenic potential, their impaired regenerative functions, fibrosis-promoting tendencies, and altered molecular profiles present significant hurdles for their direct autologous application. These limitations suggest potential advantages for allogeneic MSC-based therapies or approaches targeting the underlying profibrotic molecular pathways, such as miRNA modulation, to optimize therapeutic outcomes in SSc [79]. The main preclinical studies regarding the use of ADSCs in SSc models are summarized in Table 1.

## 4. Clinical Applications of ADSCs in SSc Treatment

Fat grafting is considered a promising treatment for microstomia, fibrosis, and other conditions that occur in scleroderma patients. By transplanting autologous fat containing ADSCs directly into the affected tissues, such as the oral and perioral regions or the hands, the procedure can enhance tissue volume and elasticity, improve function, and reduce stiffness in patients with SSc [85]. ADSCs’ anti-fibrotic properties help in reducing tissue thickening, a common issue in SSc. Furthermore, growing evidence suggests that lipotransfer can reduce collagen deposition, decrease dermal thickening, and promote angiogenesis in fibrotic conditions. ADSCs are thought to support vessel formation by secreting angiogenic factors such as insulin growth factor, VEGF, and PDGF and may also differentiate into endothelial cells, contributing directly to vascular structures [86].

The use of the patient’s own fat minimizes adverse reactions and yields natural-looking, long-lasting results. However, there are limitations: in advanced cases, repeat treatments may be required, and some of the grafted fat may be reabsorbed by the body over time, leading to variable outcomes. Each case should be individually evaluated, as the severity of fibrosis and microstomia differs between patients. Overall, fat grafting offers a viable option for enhancing facial mobility and esthetics in scleroderma-related microstomia [87]. The results of the clinical studies investigating ADSCs in SSc are summarized in Table 2.

A 2013 study described six cases of localized scleroderma, and it was one of the first to explore the therapeutic potential of ADSCs specifically for treating skin fibrosis in this condition. While the findings may not be directly extrapolated, they offer relevant insights into ADSC therapy. This research assessed the clinical effects of ADSCs by isolating and expanding them from the patients’ own adipose tissue, which were then transplanted in a hyaluronic acid (HA) scaffold to improve skin elasticity and reduce fibrosis in targeted areas. Six patients with facial and extremity involvement received autologous ADSC transplants in HA, and outcomes were assessed over a one-year follow-up period. Results showed a significant reduction in skin tightness and enhanced elasticity in treated areas, with no adverse reactions or complications. Patients reported improved cosmetic appearance and mobility, and ultrasound confirmed structural skin changes consistent with decreased fibrosis [88]. Lipofilling has been shown not only to reduce fibrosis but also to alleviate pain, as measured by a visual analog scale, and to increase capillary density, as assessed through nailfold videocapillaroscopy. These findings highlight the regenerative capabilities of ADSCs, which promote angiogenesis and modulate local inflammation, thereby supporting tissue repair. Autologous fat grafting has proven to be an effective treatment for digital ulcers in SSc patients, underscoring the potential of ADSCs in enhancing vascular regeneration and facilitating wound healing in ischemic conditions [89].

### 4.1. Lipofilling and Expanded ADSCs

ADSCs can be administered as part of the lipoaspirate in the lipofilling procedure or isolated, cultivated, and injected as a single-cell preparation. Lipofilling is easy and low cost and can be performed even under local anesthesia. It does not need specific equipment or facilities and can be easily repeated. However, it is hard to standardize, the graft-take rate is unpredictable, and often multiple procedures are needed. Cultured ADSCs offer several theoretical advantages: they allow for the transplantation of only the targeted cells in the precise numbers needed, and their expansion addresses the issue of low cell yields—particularly in lean patients with limited fat tissue. Additionally, this approach could facilitate the standardization of the procedure and help establish a clear dose/efficacy relationship. However, the need for a Good Manufacturing Practice (GMP) facility significantly increases the costs. Furthermore, the lengthy and time-intensive nature of the process, and the inability to complete both fat harvesting and graft enrichment in a single surgical session, seem to counterbalance its potential benefits, thereby limiting the widespread application of this technique [92]. Some studies have tried to compare the efficacy of isolated ADSCs and lipofilling.

In 2016, an Italian clinical study investigated the effects of fat transplantation versus adipose-derived stromal cell (ADSC) injections in improving mouth functional disability in patients with systemic sclerosis (SSc). Results showed significant improvements in both treatment groups, with increased maximal mouth opening (MMO) and reduced Mouth Handicap in Systemic Sclerosis Scale (MHISS) scores, indicating better mouth function. Satisfaction levels, evaluated using a visual analog scale (VAS), were high in both groups. However, no statistically significant difference emerged between the fat transplantation and ADSC injection groups, suggesting that both methods effectively address SSc-induced mouth disability [93].

A recent Chinese study reported that using fat grafting instead of concentrated ADSCs has shown reduced efficacy, highlighting that the active component in fat grafting is predominantly the ADSCs. This underscores the crucial role of ADSCs in mediating the regenerative and therapeutic effects observed in fat grafting, including their ability to promote angiogenesis, modulate inflammation, and support extracellular matrix remodeling. These findings suggest that isolating and concentrating ADSCs may enhance therapeutic outcomes, particularly in conditions requiring targeted cellular interventions [94]. This has been demonstrated in various applications, including in the treatment of scleroderma.

### 4.2. Fat Grafting Enrichment

Many authors have proposed the enrichment of lipotransfer to increase its efficacy. Different strategies have been described in the literature including the use of platelet-rich plasma (PRP), SVF, isolated ADSCs, or pharmacological agents.

Combining ADSCs with platelet-rich plasma (PRP) has proven to be an effective approach in treating fibrotic facial areas. The microfat grafting technique, which delivers small volumes of autologous fat enriched with PRP, has shown significant benefits. Patients treated with this method experienced enhanced skin flexibility, greater mouth opening, and improved facial contour. The combined effects of ADSCs and PRP stimulate capillary network formation and support extracellular matrix remodeling within fibrotic tissues, effectively reducing rigidity and restoring facial mobility. This innovative approach underscores the potential of regenerative therapies in managing fibrosis and enhancing functional and esthetic outcomes [42].

The rationale behind this combination lies in the regenerative properties of ADSCs in the SVF, which support tissue repair through mechanisms such as angiogenesis, immunomodulation, and extracellular matrix remodeling. PRP, on the other hand, provides a concentrated source of growth factors like platelet-derived growth factor (PDGF) and transforming growth factor-beta (TGF-β), which can further stimulate tissue repair and enhance ADSCs’ regenerative actions. Results demonstrated substantial improvements in skin elasticity and an increase in mouth opening. Ultrasound imaging showed increased capillary density in the treated areas, suggesting enhanced vascularization, a critical factor in reducing ischemic symptoms and supporting healthy tissue function. Patients reported significant esthetic and functional improvements, with a greater ease of movement and a softer skin texture in the treated areas. These findings underscore the potential of the combined SVF-PRP treatment to modulate fibrotic processes in SSc, with ADSCs promoting new blood vessel formation and collagen remodeling while PRP’s growth factors accelerate repair processes. This dual therapy approach could be a valuable, minimally invasive option for managing localized fibrosis in SSc, specifically addressing the challenging manifestations of facial involvement [90].

Stem cell-enriched lipotransfer significantly improves orofacial fibrosis in patients with SSc, addressing both functional and esthetic impairments. Using the Mouth Handicap in Systemic Sclerosis Scale (MHISS) to evaluate mouth function, studies have shown significant improvements, including reductions in MHISS scores, enhanced esthetic outcomes, and improved psychological well-being [65].

A recent paper highlighted the superiority of adipose-derived stem cell (ADSC)-assisted fat grafting over traditional fat grafting and stromal vascular fraction (SVF)-assisted grafting in treating facial atrophy caused by localized scleroderma (LoS). ADSC-assisted fat grafting demonstrated a significantly higher fat retention rate (49.83%) after six months compared to SVF-assisted (31.75%) and conventional fat grafting (21.86%). In comparison, the SVF-assisted group showed no significant improvement in fat retention compared to the conventional group at the 3-month mark. However, over the longer term, the SVF-assisted group demonstrated better fat retention, with noticeably higher levels than the conventional group at 6 months [91].

## 5. Conclusions

Current evidence suggests that ADSCs may be beneficial in SSc treatment, particularly for improving oral and facial functions through fat grafting. However, larger studies with robust designs are needed to confirm their efficacy in other disease manifestations. Moreover, the influence of the SSc environment on ADSC behavior underscores the importance of advancing our understanding of ADSC biology within the specific disease context, particularly focusing on factors that drive differentiation toward either regenerative or profibrotic outcomes. Continued research on the modulation of ADSCs and their interactions with the SSc microenvironment will be essential in refining cell-based therapies and maximizing their safety and effectiveness for treating systemic sclerosis.

## Figures and Tables

**Table 1:** Main preclinical studies evaluating the effects of adipose-derived stem cells (ADSCs.) on systemic sclerosis (SSc). Abbreviations: PBMC (peripheral blood mononuclear cells), BM-MSC (bone marrow mesenchymal cells), LMWH (low-molecular-weight heparin), and HMVEC-dA (human microvascular endothelial cells).

Authors and Year of Publication	Effects of ADSCs	Experimental Settings	Experimental Model	Reference
Higginbotham S. et al., 2024	Inhibit myofibroblast activation and reduce collagen production by downregulating the TGF-β/Smad pathway	In vitro	TGFβ1-induced model of myofibroblast differentiation was used to test the effect of conditioned media from adipose tissues, ADSCs, or lipids on the proportion of fibroblasts and myofibroblasts.	[56]
Rozier P. et al., 2021	Inhibit profibrotic pathways	In vitro	Extracellular vesicles were injected in the HOCl-induced SSc model.	[58]
Kornicka K. et al., 2018	Reduce T lymphocyte proliferation	In vitro	Azacitidine/Resveratrol-treated ADSC isolated from metabolic syndrome horses.	[60]
Kuca-Warnawin E. et al., 2022	Decrease T-cell proliferation	In vitro	PBMC and purified CD4+ T cells of healthy donors were activated and co-cultured with untreated or cytokine licensed ASCs, then analyzed by flow cytometry.	[63]
Okamura A. et al., 2020	Decrease the infiltration of CD4+ and CD8+ T cells and macrophages into the skin	Animal model (mouse)	ADSCs were intravenously administered to a scleroderma model.	[64]
Maria A.T. et al., 2016	Increase in the MMP1/TIMP1 ratio	Animal model (mouse)	Scleroderma mice received intravenous injection of BM-MSC from syngeneic BALB/c or allogeneic C57BL/6 mice and xenogeneic hBM-MSC or hADSC.	[67]
Chen W. et al., 2017	Reduced skin thickness and hydroxyproline content	Animal model (mouse)	ADSCs were subcutaneously injected into the dorsal area in the model treatment mice group.	[69]
Suzuka T. et al., 2022	Reduced skin fibrosis	Animal model	ADSCs activated with LMWH in the treatment of fibrosis.	[70]
Velier M. et al., 2019	SSc-ADSCs maintain pro-angiogenic and anti-fibrotic paracrine effects	In vitro	ADSCs were co-cultured in transwell inserts with HMVEC-dA.	[74]
Rozier P. et al., 2021	EVs have been shown to be more effective than the cells themselves in exerting anti-fibrotic effects	In vitro	ADSCs and EVs on TGFβ1-activated fibroblasts.	[75]
Rozier P. et al., 2021	EVs alleviate systemic sclerosis via miR-29a-3p	Animal model	EVs were injected in the HOCl-induced SSc murine model.	[58]

**Table 2:** Main clinical studies evaluating the effects of adipose-derived stem cells (ADSCs.) on systemic sclerosis (SSc). Abbreviation: Mouth Handicap in Systemic Sclerosis Scale (MHISS).

Authors and Year of Publication	Role of ADSC	Type of Study	Study Design	Study Results	Reference
Scuderi N. et al., 2013	Reduction in skin tightness and enhanced elasticity in treated areas	Prospective cohort study	Autologous ADSCs were transplanted into patients with scleroderma using a hyaluronic acid solution as the delivery medium	ADSC in hyaluronic acid solution determined a significant improvement in tightening of the skin without complications	[88]
Gheisari M. et al., 2018	Reduced microstomia, improving oral function and reducing stiffness	Open-label study	Autologous ADSCs were transplanted	Improvement in mouth opening capacity 3 months after autologous fat transfer	[85]
Almadori A. et al., 2019	Reductions in MHISS scores and enhanced esthetic outcomes	Open cohort study	Autologous ADSC-enriched lipotransfer treatment	Improvement of mouth function and facial volumetric appearance with improved psychological outcome	[65]
Del Papa N. et al., 2019	To alleviate pain and to increase capillary density	Case–control study	Adipose tissue grafting consisted of injection, at the base of the finger with the ischemic digital ulcer, of 0.5–1 mL of adipose tissue after centrifugation of fat aspirate	Ischemic digital ulcer healing and pain reduction were observed	[89]
Daumas A. et al., 2020	Enhanced skin flexibility, greater mouth opening, and improved facial contour	Case report	Microfat was mixed with PRP in an 80/20 proportion using two 10 mL syringes connected	Improvement in MHISS score	[42]
Virzì F. et al., 2017	New blood vessel formation and collagen remodeling	Prospective cohort study	ADSC and PRP	Improved buccal’s rhyme, skin elasticity, and vascularization	[90]
Jeon F.H.K. et al., 2020	Fat grafts appear to enhance capillary networks in the fibrotic areas	Case report	Autologous fat grafting and z plasty	Maximal mouth opening increase	[40]
Wang C. et al., 2021	Superiority of ADSC-assisted fat grafting over traditional fat grafting and SVF-assisted grafting in treating facial atrophy	Pilot study	ADSC-assisted fat grafting, fat grafting, and SVF-assisted grafting	Improvement of facial atrophy caused by LoS	[91]

## Data Availability

No new data were created or analyzed in this study.
